# Oleic Acid Increases Synthesis and Secretion of VEGF in Rat Vascular Smooth Muscle Cells: Role of Oxidative Stress and Impairment in Obesity

**DOI:** 10.3390/ijms140918861

**Published:** 2013-09-13

**Authors:** Gabriella Doronzo, Michela Viretto, Cristina Barale, Isabella Russo, Luigi Mattiello, Giovanni Anfossi, Mariella Trovati

**Affiliations:** Internal Medicine and Metabolic Disease Unit, Department of Clinical and Biological Sciences of the University of Turin, San Luigi Gonzaga Hospital, Orbassano (Turin) 10043, Italy; E-Mails: gabriella.doronzo@unito.it (G.D.); michela.viretto@unito.it (M.V.); cristina.barale@unito.it (C.B.); isabella.russo@unito.it (I.R.); luigi.mattiello@unito.it (L.M.)

**Keywords:** obesity, vascular smooth muscle cells, vascular endothelial growth factor, oleic acid, oxidative stress, protein kinase C, Zucker rats, mitogen activated protein kinase, phosphatidylinositol 3-kinase

## Abstract

Obesity is characterized by poor collateral vessel formation, a process involving vascular endothelial growth factor (VEGF) action on vascular smooth muscle cells (VSMC). Free fatty acids are involved in the pathogenesis of obesity vascular complications, and we have aimed to clarify whether oleic acid (OA) enhances VEGF synthesis/secretion in VSMC, and whether this effect is impaired in obesity. In cultured aortic VSMC from lean and obese Zucker rats (LZR and OZR, respectively) we measured the influence of OA on VEGF-A synthesis/secretion, signaling molecules and reactive oxygen species (ROS). In VSMC from LZR we found the following: (a) OA increases VEGF-A synthesis/secretion by a mechanism blunted by inhibitors of Akt, mTOR, ERK-1/2, PKC-beta, NADPH-oxidase and mitochondrial electron transport chain complex; (b) OA activates the above mentioned signaling pathways and increases ROS; (c) OA-induced activation of PKC-beta enhances oxidative stress, which activates signaling pathways responsible for the increased VEGF synthesis/secretion. In VSMC from OZR, which present enhanced baseline oxidative stress, the above mentioned actions of OA on VEGF-A, signaling pathways and ROS are impaired: this impairment is reproduced in VSMC from LZR by incubation with hydrogen peroxide. Thus, in OZR chronically elevated oxidative stress causes a resistance to the action on VEGF that OA exerts in LZR by increasing ROS.

## 1. Introduction

Obesity is characterized by increased cardiovascular morbidity and mortality [1–3]. Metabolic and vascular insulin resistance, pro-thrombotic trend, low-grade chronic inflammation, endothelial and platelet dysfunction and oxidative stress, frequently associated in a cluster, represent the main links between obesity and atherothrombosis [4–10].

Furthermore, obese subjects present a reduced development of post-ischemic collateral vessels [11], a phenomenon that increases the severity of clinical manifestation of atherosclerosis, such as sudden death, acute myocardial infarction, angina pectoris, stroke and severe peripheral ischemia [12–16]. Also type 2 diabetes is characterized by poor collateral vessel development [17]: since the large majority of type 2 diabetic patients are obese, obesity is certainly involved in this phenomenon.

Finally, the classical animal model of genetic obesity and metabolic syndrome, the obese Zucker rat (OZR), characterized by a homozygous mutation in the leptin receptor [18], shows an impaired formation of collateral vessels [19–22]. Collateral vessel formation is due to “arteriogenesis”, a complex sequence of events regulated by many molecules [23] with a pivotal role for vascular endothelial growth factor (VEGF) [23,24] and involving different vascular cells, with a pivotal role for vascular smooth muscle cells (VSMC) [25].

We previously demonstrated that human and rat aortic VSMC synthesize and secrete VEGF-A, one of the five members of the VEGF family [24], and that this phenomenon is enhanced by insulin via both phosphatidylinositol 3-kinase (PI3-K)/Akt and mitogen activated protein kinase (MAPK) pathways [26] and by high glucose via an osmotic mechanism [27]. We also showed that the increase of VEGF-A induced by insulin and high glucose is blunted in VSMC from OZR [26,27].

Interestingly, the poor formation of collateral vessels in OZR has been ascribed to reduced VEGF synthesis and to increased oxidative stress [19,21,22]. It is therefore important to further explore the links between obesity and VEGF in VSMC. Adipose tissue, which is expanded in obesity, is a dynamic endocrine/paracrine organ able to synthesize and secrete bioactive peptides, cytokines and free fatty acids, involved in metabolic and vascular homeostasis [28–31]. In this work we focused our attention on oleic acid since it is a monounsaturated fatty highly represented in humans both in plasma and in adipose tissue [32] and a main component of olive oil, a relevant constituent of the Mediterranean diet [33]; thus, its biological role has been investigated. In VSMC, oleic acid increases migration and/or proliferation [34–43] both *per se* and by enhancing the activity of angiotensin II [38,39], endothelin-1 [40], insulin-like growth factor-1 [41] and the adipocyte-conditioned medium [42,43]. As far as VSMC apoptosis is concerned, it has been recently demonstrated that oleic acid exerts an anti-apoptotic effect *per se* and dose-dependently reduces the pro-apoptotic properties of palmitic and stearic acid [44]. Furthermore, in human VSMC it increases VEGF secretion and markedly enhances the similar effect exerted by the adipocyte conditioned medium [42]. It is unknown whether in VSMC oleic acid also increases VEGF synthesis, the signaling pathways involved, and whether its effects are preserved in obesity, characterized by reduced formation of collateral vessels [11,19–22]. The aim of the present study is to clarify whether oleic acid influences VEGF-A synthesis and secretion in aortic VSMC from lean Zucker rats (LZR) and OZR, the signalling pathways involved and the role of oxidative stress.

## 2. Results

### 2.1. Time- and Concentration-Dependence of the Oleic Acid Effects on VEGF-A mRNA Transcription and on VEGF-A Protein Synthesis and Secretion in VSMC from LZR and OZR

**Figure 1:**
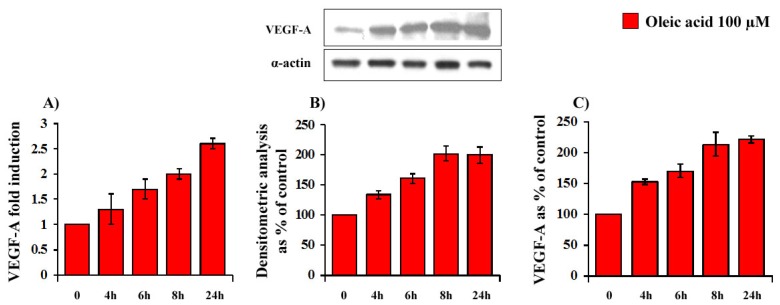
Time-dependent (4–24 h of incubation with 100 μM oleic acid) influence of oleic acid on VEGF-A mRNA transcription (Panel **A**); protein synthesis (Panel **B**) and secretion (Panel **C**) in VSMC from LZR.

Oleic acid time-dependently (4–24 h) increased VEGF-A mRNA transcription (ANOVA, *p* = 0.0001), protein synthesis (ANOVA, *p* = 0.005) and secretion (ANOVA, *p* = 0.0001) in VSMC from LZR (Figure 1 Panels A–C).

**Figure 2:**
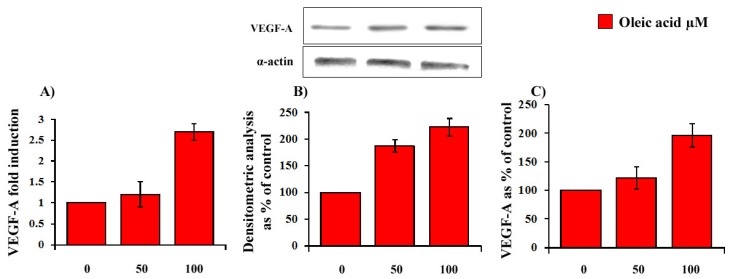
Dose-dependent (24 h of incubation with 50–100 μM oleic acid) influence of oleic acid on VEGF-A mRNA transcription (Panel **A**); protein synthesis (Panel **B**) and secretion (Panel **C**) in VSMC from LZR.

Oleic acid dose-dependently (50–100 μM, 24 h) increased VEGF-A mRNA transcription (ANOVA, *p* = 0.002), protein synthesis (ANOVA, *p* = 0.0001) and secretion (ANOVA, *p* = 0.0001) in VSMC from LZR (Figure 2 Panels A–C).

**Figure 3:**
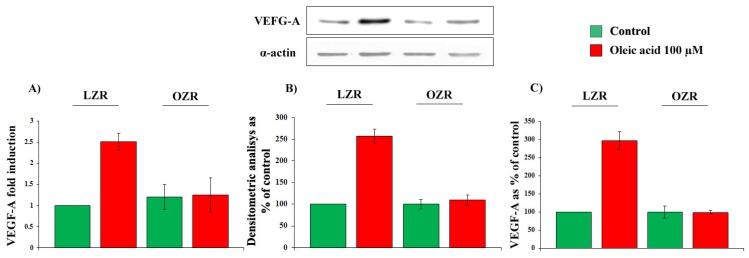
Influence of a 24-h incubation with 100 μM oleic acid on VEGF-A: (**A**) mRNA transcription; (**B**) protein synthesis and (**C**) secretion in VSMC from LZR and OZR.

As shown in Figure 3, a 24 h incubation with 100 μM oleic acid increased VEGF-A mRNA transcription (*p* = 0.0001, Panel A), protein synthesis (*p* = 0.0001, Panel B) and secretion (*p* = 0.0001, Panel C) in VSMC from LZR but not from OZR. Baseline values of VEGF mRNA transcription, protein synthesis and secretion did not differ in VSMC from LZR and OZR (Figure 3, Panels A–C).

### 2.2. Role of PI3-K and MAPK Pathways in the Increase of VEGF-A Synthesis and Secretion Induced by Oleic Acid in VSMC from LZR

**Figure 4:**
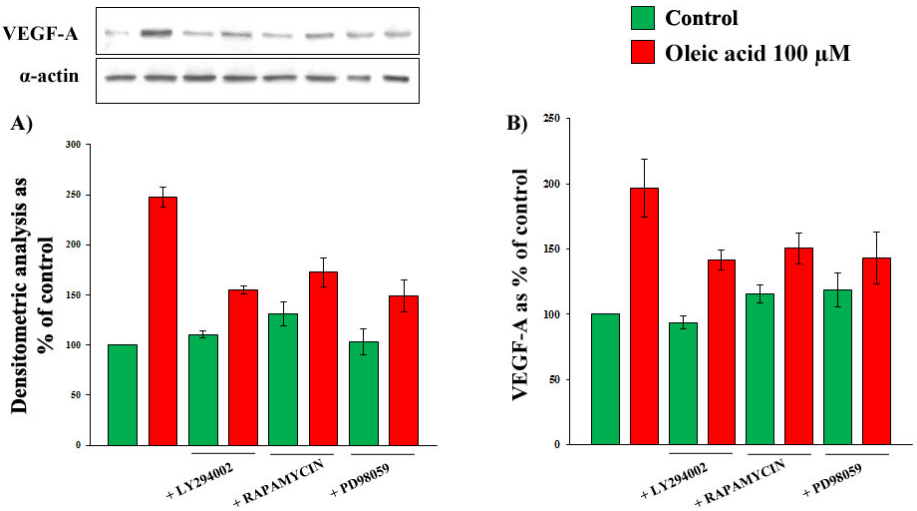
Influence of a 24-h incubation with 100 μM oleic acid on VEGF-A synthesis (Panels **A**) and secretion (Panels **B**) in VSMC from LZR, without or with a 1-h pre-incubation with inhibitors of: (i) Akt (LY294002); (ii) mTOR (Rapamycin); (iii) MEK (PD98059). All the inhibitors impair the oleic acid effects (*n* = 6, *p* = 0.04–0.02 *vs.* oleic acid alone).

As shown in Figure 4, Panels A and B, in the presence of the specific inhibitors of molecules of PI3-K and MAPK pathways, the oleic acid induction of VEGF-A synthesis and secretion in VSMC from LZR was blunted or significantly reduced (*p* = 0.02–0.04 *vs.* oleic acid alone), suggesting that these signalling molecules are involved in the oleic acid effects. Inhibitors of JNK and p38 MAPK slightly reduced, but did not inhibit the oleic-acid induced increase of VEGF-A synthesis and secretion. In particular, the values, expressed as percent of control values, are: (i) for VEGF-A protein synthesis, 222.6 ± 12.7 with oleic acid alone, 184.1 ± 9.6 with oleic acid + SP600125 and 177.8 ± 8 with oleic acid + SB203580 (*p* = 0.03–0.01); (ii) for VEGF-A secretion, 196.7 ± 15.2 with oleic acid alone, 153.3 ± 6.9 with oleic acid + SP600125 and 149.5 ± 11.3 with oleic acid + SB203580 (*p* = 0.03–0.02).

### 2.3. Role of PKC, and of PKC-Beta in Particular, in the Increase of VEGF-A Synthesis and Secretion Induced by Oleic Acid in VSMC from LZR

**Figure 5:**
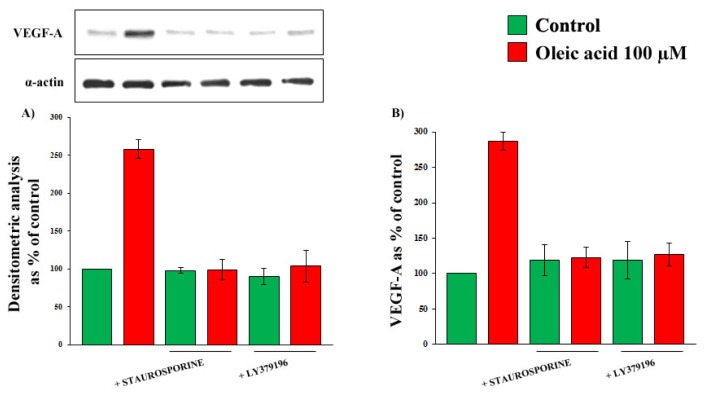
Influence of a 24-h incubation with 100 μM oleic acid (on VEGF-A synthesis (Panels **A**) and secretion (Panels **B**) in VSMC from LZR, without or with a 1-h pre-incubation with inhibitors of PKC (Staurosporine and LY379196).

As shown in Figure 5, Panels A and B, in the presence of inhibitors of protein kinase C (PKC) and of PKC-beta in particular, the increase of VEGF-A synthesis and secretion induced by oleic acid in VSMC from LZR was completely blunted (ns *vs.* baseline, *p* = 0.0001 *vs.* oleic acid for both).

### 2.4. Role of Reactive Oxygen Species (ROS) in the Increase of VEGF-A Synthesis and Secretion Induced by Oleic Acid in VSMC from LZR

**Figure 6:**
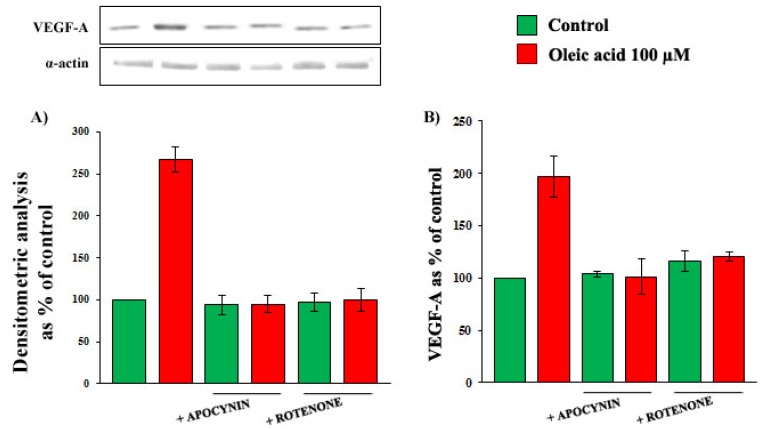
Influence of a 24-h incubation with 100 μM oleic acid on VEGF-A synthesis (Panels **A**) and secretion (Panels **B**) in VSMC from LZR, without or with a 1-h pre-incubation with inhibitors of: (i) NADPH oxidase (Apocynin); (ii) mitochondrial electron transport chain complex (Rotenone). All the inhibitors impair the oleic acid effects (*n* = 6, *p* = 0.04–0.02 *vs.* oleic acid alone).

As shown in Figure 6, Panels A and B in the presence of specific inhibitors of nicotinamide adenine dinucleotide phosphate (NADPH) oxidase and of mitochondrial electron transport chain complex, the increase of VEGF-A synthesis and secretion induced by oleic acid in VSMC from LZR was completely blunted (ns *vs.* baseline, *p* = 0.0001 *vs.* oleic acid for both).

On the contrary, xantine oxidase inhibition did not modify the oleic acid effects on VEGF-A synthesis and secretion (*p* = ns *vs.* baseline and *vs.* oleic acid alone) (data not shown).

### 2.5. Oleic Acid Signalling in VSMC from LZR and OZR

Oleic acid increased phosphorylation of Akt, p70S6K, ERK-1/2 and PKC-beta in VSMC from LZR in a time- and dose-dependent manner: the oleic acid effect was significant starting from the concentration of 50 μM and from 2 h of incubation for all the molecules (ANOVA, *p* = 0.0001, data not shown). We did not observe significant increases of JNK-1/2 and p38 MAPK phosphorylation.

**Figure 7:**
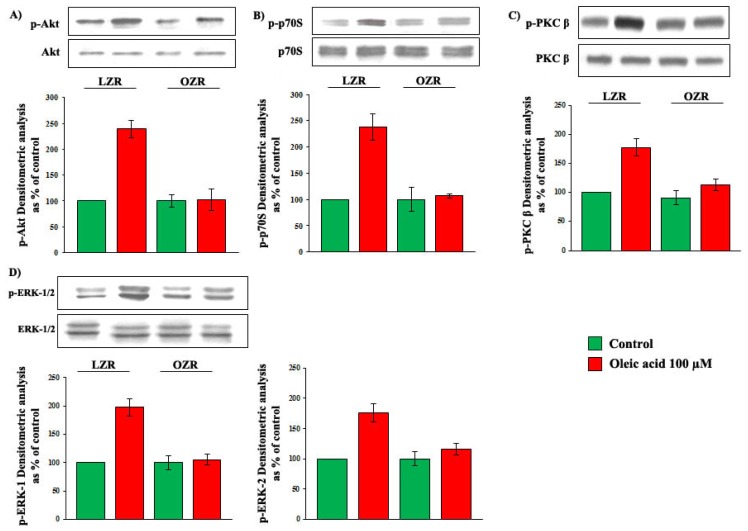
Influence of a 6-h incubation with 100 μM oleic acid on phosphorylation of Akt (Panel **A**); p70S6K (Panel **B**); ERK-1/2 (Panel **C**) and PKC-beta (Panel **D**) in VSMC from LZR and OZR. Baseline values do not differ between LZR and OZR.

Results obtained with 6 h incubation with 100 μM oleic acid are shown in Figure 7. Oleic acid significantly increased phosphorylation of Akt (Panel A), p70S6K (Panel B), ERK-1/2 (Panel C) and PKC-beta (Panel D) (*p* = 0.0001 for all the molecules) in VSMC from LZR, but not from OZR (*p* = ns *vs.* baseline). Baseline values of both non-phosphorylated and phosphorylated signalling molecules did not differ in VSMC from LZR and OZR.

**Figure 8:**
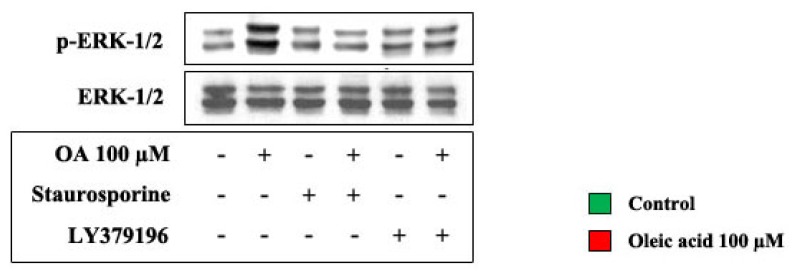

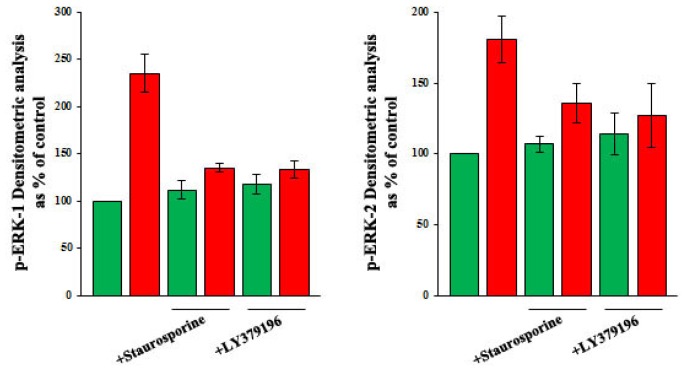
Influence of a 6-h incubation with 100 μM oleic acid on ERK-1/2 phosphorylation in VSMC from LZR, without or with a 1-h pre-incubation with inhibitors of PKC (Staurosporine and LY379196).

The influence of oleic acid on ERK-1/2 phosphorylation in VSMC from LZR was deeply impaired by inhibitors of PKC, PKC-beta. On the contrary, the oleic acid-induced activation of the PI3-K pathway was not influenced by PKC inhibition. In particular Akt values, expressed as percent of control, were: 239.8 ± 11 with oleic acid alone, 204.3 ± 16 with oleic acid + staurosporine and 217.6 ± 23 with oleic acid + LY379196 (ns *vs.* oleic acid alone) (Figure 8).

**Figure 9:**
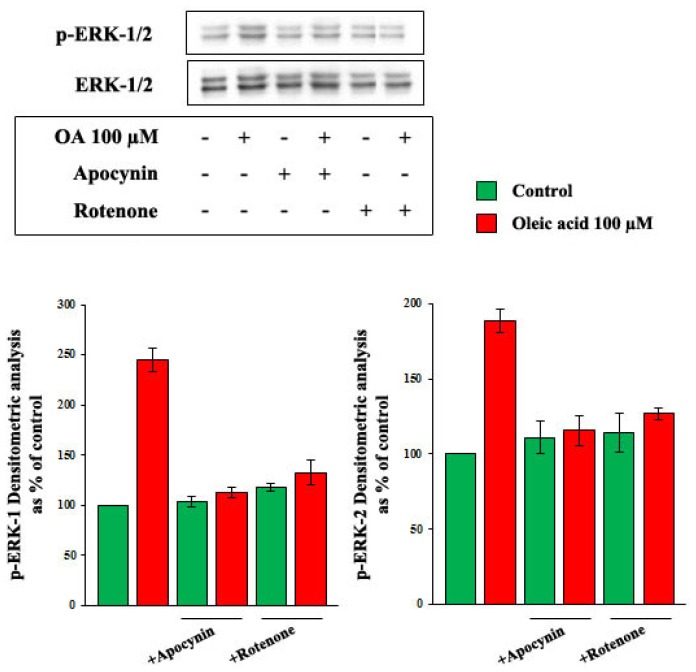
Influence of a 6-h incubation with 100 μM oleic acid on ERK-1/2 phosphorylation in VSMC from LZR, without or with a 1-h pre-incubation with inhibitors of: (i) NADPH oxidase (Apocynin); (ii) mitochondrial electron transport chain complex (Rotenone).

The influence of oleic acid on ERK-1/2 phosphorylation in VSMC from LZR was also deeply impaired by inhibitors of NADPH oxidase and mitochondrial electron transport chain complex (*p* = 0.03–0.002 *vs.* oleic acid alone) (Figure 9).

### 2.6. Oleic Acid-Induced Increase of Superoxide Anion in VSMC from LZR and OZR: Influence of PKC, NADPH Oxidase and Mitochondrial Electron Transport Chain Complex Inhibition

**Figure 10:**
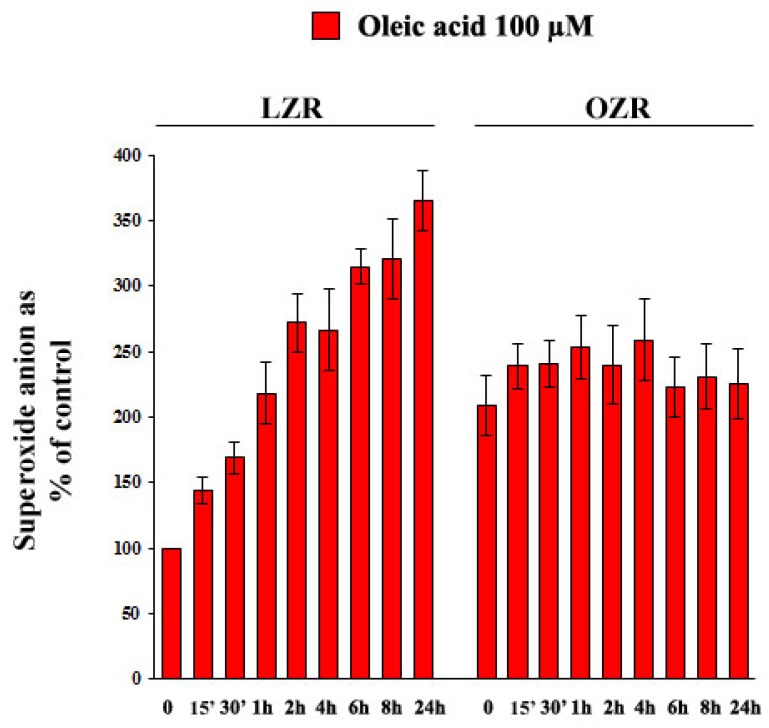
Time-dependent (15 min–24 h of incubation) influence of 100 μM oleic acid on superoxide anion in VSMC from LZR and OZR. Baseline values are greater in OZR than in LZR (*n* = 6, *p* = 0.002). Oleic acid effects are significant in LZR (*n* = 6, ANOVA, *p* = 0.0001 *vs.* baseline) but not in OZR.

As shown in Figure 10: (i) baseline superoxide anion concentrations were greater in VSMC from OZR than from LZR (*p* = 0.002); (ii) oleic acid time-dependently increased superoxide anion production in VSMC from LZR (ANOVA, *p* = 0.0001), but not from OZR (*p* = ns).

**Figure 11:**
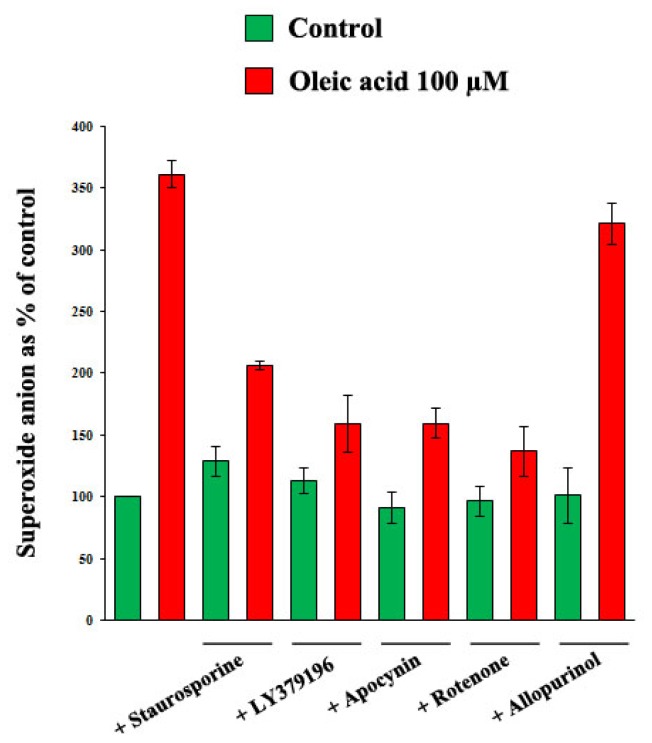
Influence of a 24-h incubation with 100 μM oleic acid on superoxide anion in LZR without or with 1-h pre-incubation with inhibitors of: (i) PKC (Staurosporine and LY379196); (ii) NADPH oxidase (Apocynin); (iii) mitochondrial electron transport chain complex (Rotenone); (iv) xantine oxidase (Allopurinol). All the inhibitors, excepting Allopurinol, impair the oleic acid effects (*n* = 6, *p* = 0.03–0.0001 *vs.* oleic acid alone).

As shown in Figure 11, the oleic acid-induced increase of superoxide anion in VSMC from LZR was impaired by inhibitors of PKC (*p* = 0.003 *vs.* oleic acid alone), of PKC-beta (*p* = 0.001 *vs.* oleic acid alone), of NADPH oxidase (*p* = 0.0001 *vs.* oleic acid alone), and of mitochondrial electron transport chain complex (*p* = 0.0001 *vs.* oleic acid alone), but not by xantine oxidase inhibition (ns both *vs.* baseline and *vs.* oleic acid alone).

### 2.7. Influence of Baseline Concentration of ROS on Oleic Acid Signalling in VSMC from LZR

**Figure 12:**
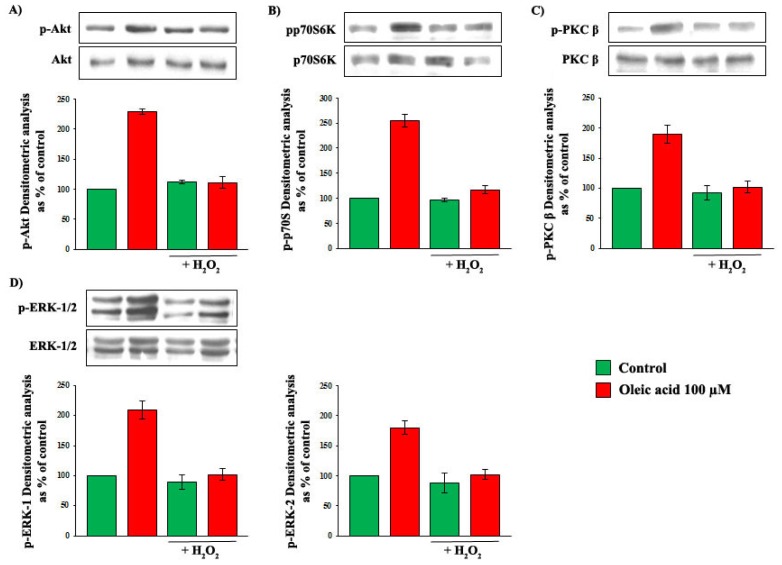
Influence of a 6-h incubation with 100 μM oleic acid on phosphorylation of Akt (Panel **A**); p70S6K (Panel **B**); ERK-1/2 (Panel **C**) and PKC-beta (Panel **D**) in VSMC from LZR without or with a 24-h pre-incubation with H_2_O_2_. H_2_O_2_ completely blunts the oleic acid effect on signaling molecules (*n* = 6, *p* = 0.001–0.0001 *vs.* oleic acid alone).

To clarify whether the baseline increase of oxidative stress observed in VSMC from OZR played a role in the impairment of the oleic acid effects on intracellular signalling, we pre-incubated VSMC from LZR with 0.5 μM H_2_O_2_ for 24 h, and we observed that this pre-incubation completely blunted the oleic acid-induced activation of PI3-K, MAPK and PKC pathways (Figure 12, Panels A–D) (*p* = 0.001–0.0001).

### 2.8. Influence of Baseline Concentration of ROS on the Increase of VEGF-A Synthesis and Secretion Induced by Oleic Acid in VSMC from LZR

**Figure 13:**
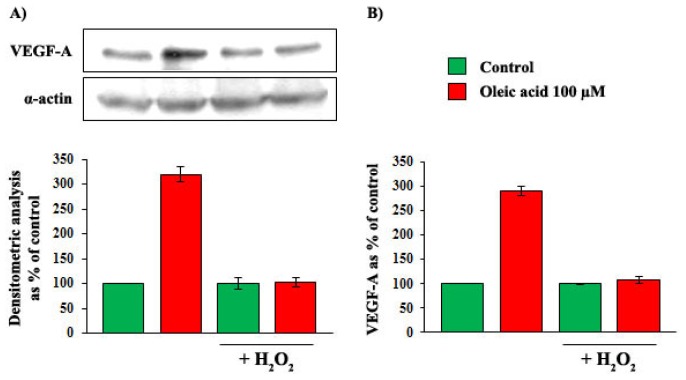
Influence of a 6-h incubation with 100 μM oleic acid on VEGF-A synthesis (Panel **A**) and secretion (Panel **B**) in VSMC from LZR without or with a 24-h pre-incubation with H_2_O_2_. H_2_O_2_ completely blunts the above mentioned effects of oleic acid on VEGF-A (*n* = 6, *p* = 0.0001 *vs.* oleic acid alone).

To clarify whether the baseline increase of oxidative stress observed in VSMC from OZR played a role in the impairment of the oleic acid effects on VEGF-A synthesis and secretion, we pre-incubated VSMC from LZR with 0.5 μM H_2_O_2_ for 24 h, and we observed that this pre-incubation completely blunted the oleic acid-induced increase of VEGF-A synthesis and secretion (Figure 13, Panels A and B) (*p* = 0.0001).

## 3. Discussion

The present study shows that oleic acid increases in a concentration- and time-dependent manner VEGF-A mRNA expression, synthesis and secretion in VSMC from LZR with a mechanism mediated by a complex network of intracellular signalling molecules, involving Akt, mTOR, ERK-1/2, PKC-beta, NADPH oxidase and the mitochondrial electron transport chain complex. We also demonstrated the ability of oleic acid to activate all the above-mentioned signalling pathways and to increase oxidative stress.

The oleic acid effect on VEGF is therefore mediated by the same pathways employed to influence VSMC proliferation and, in some studies, migration. Actually, it has been reported that oleic acid enhances VSMC proliferation by: (i) PKC and ERK activation [34]; (ii) ROS increase [35]; (iii) Akt/PKB [36] and mTOR activation [42]. It is also able to enhance VSMC migration via PKC/ERK activation and via oxidative stress [39].

Our results support the occurrence of the following chain of events in VSMC from LZR: (i) oleic acid activates PKC-beta; (ii) PKC-beta is involved in the oleic acid-induced increase of oxidative stress, since it is impaired by LY379196; (iii) oxidative stress is involved in the activation of ERK-1/2, since it is impaired by apocynin and rotenone; (iv) ERK-1/2 play a crucial role in the increase of VEGF-A synthesis and secretion, since these effects are impaired by PD98059.

We further demonstrate that the acute increase of ROS induced by oleic acid involves NADPH oxidase, being impaired by apocynin, and the mitochondrial electron transport chain complex, being impaired by rotenone, but does not involve xanthine oxidase, being unaffected by allopurinol. The fact that both NADPH oxidase and the mitochondrial electron transport chain are involved in the oleic acid-induced ROS generation in VSMC is in agreement with similar results obtained in beta-cells [45]. As far as we know, our work is the first one to address the effects of oleic acid on oxidative stress in VSMC. Further studies are needed to explore in detail the oleic acid effects on mitochondrial oxidation and biogenesis and on NADPH-induced ROS generation. It is known from experiments carried out in isolated aorta from LZR and OZR that in OZR both mitochondrial and cytosolic superoxide anion production is increased [22] and NADPH oxidase activity is enhanced [46]. It should be interesting to repeat in isolated aortas the experiments with oleic acid we carried out in isolated VSMC. Furthermore, additional studies are needed to clarify whether the oleic acid-induced effects on VEGF synthesis are mediated by hypoxia-inducible factor (HIF)-1a activation, since it is the major transcription factor involved in VEGF induction [24].

A further intriguing result of our study is that the effect of oleic acid on VEGF-A is impaired in VSMC from OZR, where the oleic acid-induced activation of the above-mentioned signalling molecules (*i.e.*, Akt, mTOR, ERK-1/2, PKC-beta) is deeply attenuated and where the oleic acid-induced ROS increase is absent.

Baseline concentrations of superoxide anion are significantly greater in VSMC from OZR, in agreement with our previous observations [47,48]. The present study indicates that the elevated baseline oxidative stress plays a pivotal role in the impaired action of oleic acid on intracellular signalling and on VEGF-A. Actually, the defects observed in VSMC from OZR have been reproduced in VSMC from LZR by a 24 h pre-incubation with hydrogen peroxide: in these experimental conditions, the oleic acid-induced activation of Akt, mTOR, ERK-1/2 and PKC-beta are completely blunted and, consequently, the oleic acid-induced increase of VEGF-A is absent. We are aware that incubation with hydrogen peroxide is not able to mimic the effects of superoxide anion, since both molecules exhibit different mechanisms of action: thus, the experiments carried out with hydrogen peroxide simply demonstrate that when oxidative stress is increased VSMC are insensitive to the influence of oleic acid on VEGF.

Thus, our data suggest that the acute increase of ROS induced by oleic acid plays a crucial role in the molecular mechanisms involved in VEGF synthesis and secretion in VSMC from LZR, whereas the chronically high oxidative stress impairs the ability of VSMC from OZR to respond to oleic acid with a further increase of ROS and the consequent activation of the signalling pathways involved, even if the baseline, constitutive VEGF synthesis is unaffected.

It is not surprising that a chronic increase of oxidative stress impairs mechanisms implicated in collateral vessel formation, which are activated by a rapid increase of the oxidative stress itself. Actually, in Wistar Kyoto rats an optimal concentration of ROS allows the coronary collateral growth in response to repetitive ischemia, a phenomenon blunted by inhibitors of both mitochondrial electron transport chain and NADPH oxidase [49], whereas in OZR the baseline elevated oxidative stress due to mitochondrial dysfunction abrogates the process of coronary collateral growth in response to repetitive ischemia, which is restored by the elimination of the mitochondrial oxidative stress [22].

In previous papers, we observed that the baseline increase of oxidative stress is involved in other defects occurring in VSMC from OZR [47,48]. In particular, it accounts for the impaired activation of the nitric oxide (NO)/cyclic guanosine monophosphate (cGMP)/cGMP-dependent protein kinase (PKG) pathway [47] the impaired PKG-induced stimulation of PI3-K and MAPK signalling pathways [48] and the impaired PKG-mediated increase of VEGF-A in response to insulin and NO [26].

As far as we know, we are providing here the first demonstration of the mechanisms involved in the oleic acid-induced increase of VEGF-A in VSMC and in its impairment in a classical animal model of obesity.

An intriguing aspect of our study is the demonstration of an oleic acid resistance in VSMC from OZR, in which we previously observed the occurrence of insulin resistance [26,48]. The increased production and release of free fatty acids is described in human and animal models of obesity as a consequence of insulin resistance [50]. Furthermore, oleic acid accounts for a greater proportion of the total free fatty acid serum concentration in OZR than in LZR [51]. The demonstration of a resistance to oleic acid in VSMC from OZR that we demonstrated here suggests that in obesity the oleic acid overproduction could be accompanied by a reduction of its biological effects. Furthermore, oleic acid is a component of olive oil, an important constituent of the Mediterranean diet [33], which is strongly recommended for the prevention of cardiovascular disease: our results suggest that some of the biological effects of exogenous oleic acid can differ according to the presence or absence of obesity.

Since VEGF is deeply involved in collateral vessel formation [23,52], the peculiar aspect of vascular resistance to oleic acid described in this study sheds light on the mechanisms potentially involved in the reduction of collateral vessels that has been observed *in vivo* both in human [11] and in animal obesity [19–22], a phenomenon playing a role in the pathogenesis of cardiovascular events [12–16].

## 4. Experimental Section

### 4.1. Research Design

To evaluate the time-dependent effect of oleic acid on VEGF-A mRNA transcription, protein expression and secretion, VSMC from lean, insulin sensitive (fa/+) Zucker rats (LZR) and obese, insulin resistant (fa/fa) Zucker rats (OZR) were incubated for 8 and 24 h with oleic acid (100 μM). Dose-dependent effects were investigated by incubating VSMC with 50–100 μM oleic acid for 24 h.

To clarify the role of PI3-K and MAPK pathways in the oleic acid-induced VEGF-A synthesis and secretion, VSMC from LZR were pre-incubated for 1 h with: (i) the Akt inhibitor LY294002 (50 μM), (ii) the mTOR inhibitor rapamycin (100 nM); (iii) the MEK inhibitor PD98059 (30 μM); (iv) the JNK inhibitor SP600125 (15 μM); (v) the p38 MAPK inhibitor SB203580 (10 μM).

To clarify the role of PKC in the oleic acid-induced VEGF-A synthesis and secretion, VSMC from LZR were pre-incubated for 1 h with the non-selective inhibitor of PKC staurosporine (10 nM) and with the specific inhibitor of PKC-beta LY379196 (10 nM).

To evaluate the role of ROS in the oleic acid-induced VEGF-A synthesis and secretion, VSMC from LZR were incubated for 1 h with: (i) the specific inhibitor of nicotinamide adenine dinucleotide phosphate (NADPH) oxidase apocynin (30 μM); (ii) the specific inhibitor of the mitochondrial electron transport chain complex rotenone (10 μM); (iii) the specific inhibitor of xanthine oxidase allopurinol (1 μM).

To verify the oleic acid ability to activate intracellular signaling pathways and ROS formation, VSMC from both LZR and OZR were incubated with different concentrations of oleic acid (50–100 μM) for different times (15 min–24 h) to measure: (i) the phosphorylated and non phosphorylated molecules Akt and p70S6K, belonging to the PI3-K pathway; ERK-1/2, JNK-1/2 and p38 MAPK, belonging to the MAPK pathway; PKC-beta; (ii) ROS formation in the absence and in the presence of apocynin, rotenone and allopurinol at the concentrations described above.

Furthermore, to verify whether PKC activates ROS and ROS activate MAPK pathway, VSMC from LZR and OZR were incubated with 100 μM oleic acid for 6 h to measure: (a) ROS formation in the presence of 1 hour pre-incubation with the PKC inhibitors staurosporine and LY379196; (b) ERK-1/2 activation in the presence of 1 h pre-incubation with the ROS inhibitors apocynin and rotenone or the PKC inhibitors staurosporine and LY379196. Inhibitors were used at the above-described concentrations.

To verify whether the increased baseline concentrations of ROS observed in VSMC from OZR were responsible of the impaired action of oleic acid on intracellular signalling and on VEGF-A synthesis and secretion, we repeated the above mentioned experiments in VSMC from LZR after induction of oxidative stress by a 24 h pre-incubation with H_2_O_2_ (0.5 μM).

### 4.2. Chemicals

Oleic acid, LY294002, PD98059, SP600125, SB203580, staurosporine, apocynin, rotenone and allopurinol were from Sigma-Aldrich (St. Louis, MO, USA). LY379196 was from Eli Lilly, (Indianapolis, IN, USA). Compounds used for real-time PCR, western blotting, enzyme-linked immunoadsorbent assay (ELISA) and for measurement of superoxide anion are detailed in Sections 4.4.–4.7.

Sodium salt of oleic acid was dissolved in water as 6 mM stock solution, as described elsewhere [42], and further diluted in Minimal Essential Medium (MEM) with 4% bovine serum albumin. For all the experiments with oleic acid, we carried out control experiments in which VSMC were incubated with bovine serum albumin alone.

### 4.3. Cell Culture and Characterization

Experiments were done on VSMC derived from aortas of LZR and OZR. Male Zucker rats, purchased from Charles River Laboratories Italy (Calco, Italy), were fed with standard rodent chow and water *ad libitum* and sacrificed with CO_2_ after a 12 h fast, when they were 14 weeks old: the aorta was removed immediately after.

VSMC isolation and characterization were made according to classical procedures, as previously reported [26]. VSMC were used at the 6th–7th passage and cultured in dishes until 80% confluence was achieved; then medium with 10% FCS was removed and cells were cultured overnight in medium with 0.1% bovine serum albumin, changed before experiments.

Experiments conform to the Directive 2010/63/EU of the European Parliament and are in agreement with the recommendations of our Department.

### 4.4. VEGF-A mRNA Expression

VSMC from LZR and OZR were incubated for 24 h with oleic acid (50–100 μM); then, total RNA was extracted using TRIzol Reagent (Applied Biosystems, Monza, Italy) following the manufacturer’s instructions after the incubation times indicated. cDNA was synthesized by reverse transcription (RT) from 2 μg RNA with a commercial kit (High-Capacity cDNA Reverse Transcription Kit; Applied Biosystems). Singleplex real-time polymerase chain reaction (PCR) was performed on 30 ng of cDNA using TaqMan Gene Expression Assay kits prepared for rat VEGF-A and β-actin, TaqMan Fast Universal PCR Master Mix, and 7500 Fast Real-Time PCR System (Applied Biosystems, Monza, Italy). Negative controls did not include cDNA. The oligonucleotide sequences are not revealed by the manufacturer because of proprietary interests. Results were then normalized to the expression of β-actin, as housekeeping gene. Relative quantification of target gene expression was achieved with a mathematical method proposed by Livak and Schmittgen [53].

### 4.5. VEGF-A Secretion and Protein Expression

To quantify VEGF secretion, at the end of incubation times, supernatant-conditioned medium was stored at −20 °C. VEGF-A levels were then measured by a specific ELISA kit from R & D System (Minneapolis, MN, USA). VEGF-A protein concentrations were normalized to the total amount of cell proteins contained in each dish as determined by the Bradford Reagent method (Sigma-Aldrich, St. Louis, MO, USA) and expressed as percent of control.

VEGF-A protein expression was measured by western blotting. Samples were separated by 8% sodium-dodecyl sulphate-polyacrylamide gel electrophoresis (SDS-PAGE) and transferred to Immobilon-P Transfer Membrane (Millipore Corporation, Bedford, MA USA). Membranes were incubated with monoclonal anti-VEGF (Santa Cruz Biotechnology Inc., Santa Cruz, CA, USA) in PBS containing 0.1% Tween-20 (Sigma-Aldrich, St. Louis, MO, USA) for 1 h. Blots were then incubated with an anti-mouse IgG (whole molecule) peroxidase conjugate antibody (Santa Cruz Biotechnology Inc., Santa Cruz, CA, USA) in PBS containing 0.1% Tween-20 (Sigma-Aldrich, St. Louis, MO, USA) for 45 min. VEGF protein was detected with ECL-plus kit (Amersham Pharmacia Biotech, Sunnyvale, CA, USA). As loading control, a monoclonal antibody, anti α-smooth muscle actin (Santa Cruz Biotechnology Inc., Santa Cruz, CA, USA) was used. Blots were analysed densitometrically by Kodak 1D Image Analysis Software. Density of bands was quantified as arbitrary units and changes in protein synthesis expressed as percent of control bands.

### 4.6. Activation of Akt, p70S6K, ERK-1/2, JNK-1/2, p38 MAPK and PKC

To measure non phosphorylated Akt, p70S6K, ERK-1, ERK-2, JNK-1 and JNK-2, p38 MAPK, PKC-beta and Akt phosphorylated at Ser-473, p70S6K phosphorylated at Ser-411, ERK-1/ERK-2 phosphorylated at Tyr-204, JNK-1/JNK-2 phosphorylated at Thr-183 and Tyr-185, p38 MAPK phosphorylated at Tyr-182, PKC-beta phosphorylated at Ser-660, VSMC extracts have been separated by 8% SDS-PAGE and transferred to Immobilon-P Transfer Membrane (Millipore Co., Bedford, MA, USA). Membranes have been incubated (60 min) with monoclonal and polyclonal antibodies (antibodies dilutions 1:1000–1:50000) against all the previously quoted molecules (Enzo Life Sciences AG, Lausen, Switzerland; Santa Cruz Biotechnology Inc., Santa Cruz, CA, USA and Epitomics Inc., Burlingame, CA, USA) in PBS containing 0.1% Tween-20 (Sigma-Aldrich, St. Louis, MO, USA). Then, blots have been incubated with a peroxidase-conjugate affine pure goat anti mouse IgG antibody (antibody dilution was 1:3000) (Santa Cruz Biotechnology, Inc., Santa Cruz, CA, USA) and with a peroxidase-conjugated affine pure goat anti rabbit IgG antibody (antibody dilution was 1:3000) for 45 min in PBS containing 0.1% Tween-20 (Sigma–Aldrich, St. Louis, MO, USA). Signalling molecules have been detected with ECL-plus kit (Amersham Pharmacia Biotech, Sunnyvale, CA, USA). Blots have been analysed densitometrically by Kodak 1D Image Analysis Software. Density of bands has been quantified as arbitrary units and changes in protein synthesis have been expressed as percent of control bands. Density of bands concerning phosphorylated proteins has been normalized with the density of un-phosphorylated ones.

### 4.7. Measurement of Superoxide Anion

To evaluate whether oleic acid increases oxidative stress in VSMC from LZR and OZR, we measured the concentrations of superoxide anion by a lucigenin-enhanced chemiluminescence method, based on light emission from reaction between reduced lucigenin and superoxide anion. Briefly, VSMC were resuspended at 5 × 105 cells/mL into phosphate buffer and maintained at 37 °C for 10 min. After a 5-s dark adaptation, lucigenin (final concentration 5 μM) was added into the 96 well-plate and chemiluminescence was recorded 3 s after the last lucigenin injection over a 60-min period at 1-min intervals by the luminescence reader GloMax-Multi Detection System (Promega Corporation, Madison, WI, USA). Intracellular superoxide anion levels were measured as relative light unit (RLU) and expressed as percent of control.

### 4.8. Statistical Analysis

All the experiments were carried out in sextuplicate. Data are expressed as mean ± SEM. Significance was evaluated, when appropriate, by unpaired Student’s *t*-test and by one-way ANOVA followed by the Bonferroni’s analysis. A *p* < 0.05 was considered significant.

## 5. Conclusions

In conclusion, this study demonstrates that OA increases in aortic VSMC form LZR VEGF synthesis and secretion by mechanisms involving oxidative stress, and that these OA actions are impaired in aortic VSMC from OZR, a classical animal model of obesity. These observations open new perspectives to further explore the intriguing field of the mechanisms involved in the poor formation of collateral vessels in human and animal obesity.
